# Gantry angle dependent beam control optimization of a traveling wave linear accelerator to improve VMAT delivery

**DOI:** 10.1002/acm2.13047

**Published:** 2020-10-23

**Authors:** Adriaan A. van Appeldoorn, Johannes G. M. Kok, Jochem W. H. Wolthaus

**Affiliations:** ^1^ Department of Radiotherapy University Medical Center Utrecht Utrecht The Netherlands

**Keywords:** 2T and 2R, beam physics, beam symmetry, radiotherapy, linac Lookup Table, VMAT

## Abstract

**Introduction:**

Increased modulation and dynamical delivery of external beam radiotherapy (EBRT), such as volumetric modulated arc therapy (VMAT) with dynamic gantry rotation, continuously variable dose rate (CVDR) and field shapes that change during the beam, place greater demands on the performance of linear accelerators (linac). In this study, the accuracy of the linac beam steering is improved by the application of a new method to determine the gantry‐dependent lookup table.

**Methods:**

An improved method of lookup table creation based on service graphing information from the linac is investigated. This minimizes the impact of magnetic hysteresis due to the previous current in the steering magnets, which is dependent on the previous gantry angle. A software tool, programmed with MATLAB®, is used to calculate and export the new optimal lookup table (LUT).

**Results:**

This method is efficient requiring little clinical machine time or analysis time, and leads to an improved VMAT delivery with a reduction of about 60 percent in beam steering errors. If the surrounding magnetic field is changed, for example, ramping a nearby magnetic resonance imaging system (MRI), the beam steering LUT optimization can be quickly performed.

**Conclusion:**

This study shows an improved linac stability using improved lookup tables. Resulting in a lower number of interruptions, preventing down‐time, and a lower risk of intrafraction motion due to longer treatment times.

## 1 INTRODUCTION

In the last decades, multiple new techniques like SBRT, IGRT, IMRT, and volumetric modulated arc therapy (VMAT) have been clinically implemented in radiotherapy. These new techniques require highly accurate dose depositions with an increased range of modulation. Dose modulation is achieved by multiple stacked dose segments. Radiation beam positioning and dynamic beam behavior becomes more and more important, as the modulation increases with the required complexity of the radiotherapy treatment. With a large number of small individual segments, a deviation of beam alignment has direct influence on the dose delivery, which might result in geometrical and dosimetric deviations. For safety reasons, beam interruptions occur if deviations in beam steering exceed the system tolerances. The problems might become even worse when magnetic resonance imaging (MRI) systems (or MRI‐Linacs) are installed in the radiotherapy department close to the existing linacs. Standard tooling (provided by the vendor) for compensation of the earth magnetic field in the beam steering is not intended for the complex fringe fields environments in case of the adjacent MRI systems.^1^, ^2^ This article describes an optimized beam steering to prevent beam interruptions for Elekta SL25 travelling wave linear accelerator (Elekta AB, Sweden).

**Fig. 1:**
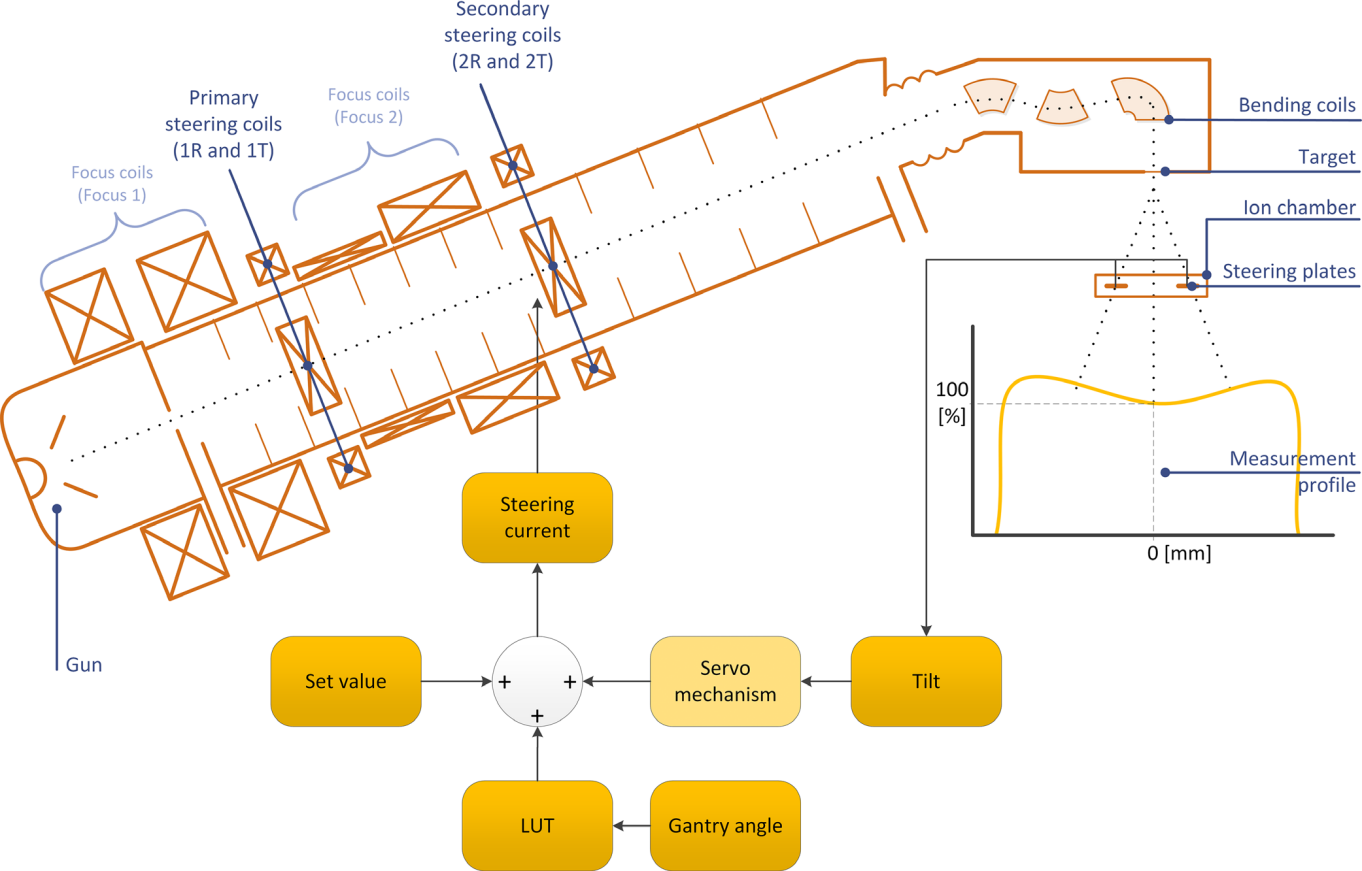
Simplified graphical overview of the steering mechanism of the traveling wave linear accelerator SL25 (Elekta AB, Sweden). Electrons coming from the filament of the electron gun will be injected into the accelerator waveguide. Two sets of primary steering coils (1R and 1T) will center the injected electron beam. The current of these primary steering coils are set for each energy. A secondary set of 2 coils (2R and 2T) are located half way down the waveguide. These coils align the electron beam to hit the target at the correct angle. With the correct current of these steering coils a symmetrical radiation beam will be produced. The actual steering currents are set by three parameters: The Set Value, a lookup table (LUT) and the output of the servo mechanism. The LUT is the initial correction to compensate for the external magnetic distortion per gantry angle. The servo mechanism controls the current based on the readout of the steering plates of the integrated ion chamber.

Figure 1 shows the schematic overview of the linac. Electrons coming from the filament of the electron gun will be injected into the accelerator waveguide. To focus the electron beam into the waveguide, two sets of focus coils are placed around the waveguide. Focus 1 before the primary steering coils (1R and 1T) and Focus 2 between the primary and secondary steering coils (2R and 2T). These focus coils cause a helical rotation of the electron trajectories. The two sets of primary steering coils will center the injected electron beam. The current of these primary steering coils are set for each energy by a static value. A secondary set of two coils are located half way down the waveguide. These coils align the electron beam to hit the target at the correct angle. With the correct current of these steering coils a symmetrical radiation beam will be produced. At the end of the waveguide a set of bending coils are used to bend the electron beam in to the direction of the target.

The beam steering settings on a linear accelerator are historical based on a flattened field measured at isocenter position. The electron beam hits a small target at the end of the electron accelerator waveguide. This creates a photon beam with a droplet shaped intensity distribution. This beam is conventionally aligned with a flattening filter which attenuates more of the central radiation in order to achieve a flat dose profile at isocenter. A misalignment of the electron beam will create an asymmetric photon radiation beam which can introduce machine errors and interruptions. This misalignment could also shift the beam spot position, the beam angle or a combination thereof.^3^, ^4^

The four sets of electron beam steering coils, known as centering coils, sets the beam inline and crossline symmetry. Crossline (IEC 61217 x) is the plane through isocenter which contains the beam target at all gantry angles. Inline (IEC 61217 y) is the vertical plane orthogonal to the crossline. Two sets of coils controls the inline direction and 2 sets of coils the crossline direction. 1T and 2T stands for the first and second steering coils of the “Transverse” movement perpendicular to the direction of bending and 1R and 2R stands for the “Radial” movement in the direction of bending. These non‐intuitive combinations of coils are due to the rotation of the electron beam, of approximately 90° caused by the focus coils. At the end of the electron beam trajectory, the inline beam position is fine‐tuned using “Bending Fine” coils. This bending fine also has a significant influence in the beam symmetry.

The radiation beam is monitored with an integrated ion chamber (IC) in the linac. This ion chamber consists of two layers for dosimetry and one to measure the spectrum of the beam. The beam symmetry is measured with two sets of plates in the ion chamber. These plates measure a tilt in symmetry as percentage of dose. For inline (“2R error”) and crossline (“2T error”) direction. To calibrate the tilt, a weighting factor (“Balance”) and a gain (“Loop”) is used. These items are described in Table 1. With the 2T and 2R Balance items the weighting factor of a beam tilt is determined, and the Loop items are used to set the gain of the error value. The gain factor is determined such that the internal error values match with profile measurements using an external QA device. There is a first‐order linear relation between the raw measured values and the error readout.

**Table 1:** Ooverview of 2R‐2T item numbers of the Integrity® software.

Item 2R	Item 2T	Description
127	128	Tilt error/plates ratio
164	165	Control current
308	310	Balance
309	311	Loop

The control mechanism for the 2R‐2T secondary beam steering current consist of three parameters:

The first parameter is a static set value of the 2R‐2T current to aim for.

Secondly, a correction for electromagnetic field per gantry angle is applied defined by a lookup table (LUT). Application of this LUT compensates the gantry angle dependency of the earth’s magnetic field. Every LUT position represents a part of the gantry arc (about 4 degree). Gantry rotation within a fixed external magnetic field results in a sinusoidal variation in the LUT. The third parameter is a servo mechanism of the secondary steering coil^1^. This servo compensates for the non‐deterministic variables using the steering current to minimize the error based on the readings of the ion chamber steering plates. Although the beam contains transient effects, the beam will become symmetrical according to the read‐out of the integrated IC.

Switching on the radiation beam, the initial 2R and 2T current are set by the Set value plus the LUT. After beam on, the IC will measure the beam tilt (symmetry deviation) and the servo system will compensate. The initial steering current is derived by Eq. (1) in which the LUT values are discretized in 2048 steps.

(1) $$I_{\mathrm{steering}}\;=\;Set\;value\;+\;LUT\;value*\frac{Calibration\;gain}{2048}.$$

If these first two parameters of the control mechanism (Set value and LUT) have sub‐optimal settings, there is an increased chance of beam interruptions during treatment due to symmetry deviations. These treatment interruptions lead to longer treatment delivery time which may cause larger intrafraction motion. Although, generally the treatment can be resumed after a reset by an authorized person (RTT, Physicist or Engineer) delays can vary from half a minute up to multiple minutes. In extreme cases multiple interruptions may lead to postponed treatment.

The aim of beam steering is to have a symmetrical radiation beam shape under all dynamic circumstances. To calibrate the linac 2T‐ and 2R beam steering parameters a beam with maximum field size is used.^5^ A QA device (2D array or water phantom) measures the beam profile at isocenter.^6^, ^7^, ^8^ To obtain a symmetric profile conditions on the QA device, the linac 2T‐ or 2R beam steering parameters will be defined.

The tilt is defined as the difference between the measured percentage doses at ±12 cm from central axis perpendicular to the beam line from the focal spot [Eq. (2)]. The dose value of the central axis is taken as the 100 percent dose reference.

(2) $$T=\frac{D\left(12\;\text{cm}\right)‐D\left(‐12\;\text{cm}\right)}{D\left(0\;\text{cm}\right)}.$$

Subsequently during clinical operation, beam symmetry is monitored by the calibrated linac ion chamber. Beam symmetry should not vary during gantry rotation. Using Elekta’s service graphing tool (Integrity® version 4), a measured plot can be acquired of the residual symmetry variation. This symmetry graph reflects the quality of the LUT. Ideally the measured tilt is close to 0% and much smaller than ±5% where the linac interrupts due to a tilt error. Note that the servo control system should be switched off while recording.

**Fig. 2:**
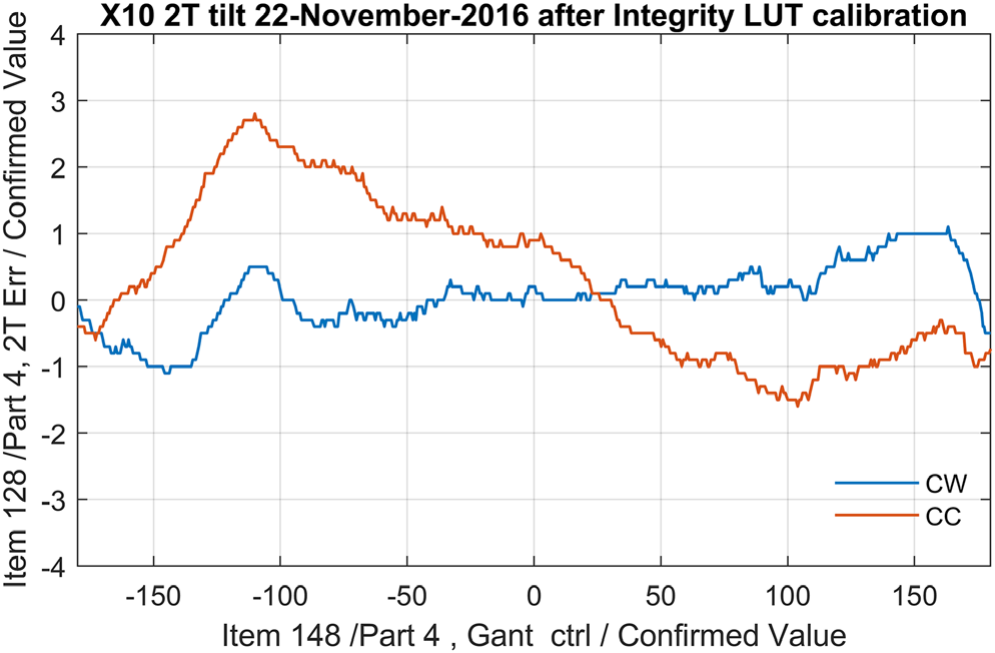
Example of gantry angle (x‐axis) dependency check of 2T tilt (y‐axis) in clock‐wise (CW) and counter clock‐wise (CC) direction after LUT optimization using Elekta procedure in CW direction. The blue line shows a good result for the CW direction. Hysteresis and system delay introduces larger deviations in the CC direction.

Although the beam tilt should be close to zero, due to hysteresis and system delays the symmetry of the beam can vary with the direction and speed of the gantry rotation. These hysteresis effect is due to the changes in current. The current will change with the LUT which is dependent on the gantry angle. Depending on how the metal has been magnetized by the previous current, it results in the effect that the magnetic field is slightly different. As shown in Fig. 2 using the default LUT creation tools from Elekta, the error readout varies up to 3% due to these effects. The LUT of this figure is made with the Elekta software using a “learn” procedure.^5^ In which the gantry slowly rotates clockwise (CW) from −183° to +183° with beam on while the servo system “learns” the gantry dependent beam steering by applying the servo mechanism feedback values.

During rotation, the last servo values of the discretized LUT positions of the gantry angles are recorded as the new LUT values. If last servo value has some spike due noise in the ion chamber readout, this noise is translated in a noise spike in the LUT. Normally, this procedure gives good results for the CW direction. However, due to hysteresis, the errors observed during counter‐clockwise (CC) rotation are greater.

To overcome these shortcomings, an improved method for LUT generation has been developed.

When a new MRI system is installed close to a linac, or up‐ or down‐ramped, a new LUT is needed to optimize the initial steering current for the influence of the fringe field of the surrounding magnetic field.^1^, ^2^ This new LUT has to be functional under clockwise (CW) and counter‐clockwise (CC) gantry rotation for all kind of arc therapies, for example, VMAT.

This article describes the developed method to improve the linac beam steering lookup tables and provides example results demonstrating a more stable radiation beam.

## 2 MATERIALS AND METHODS

**Fig. 3:**
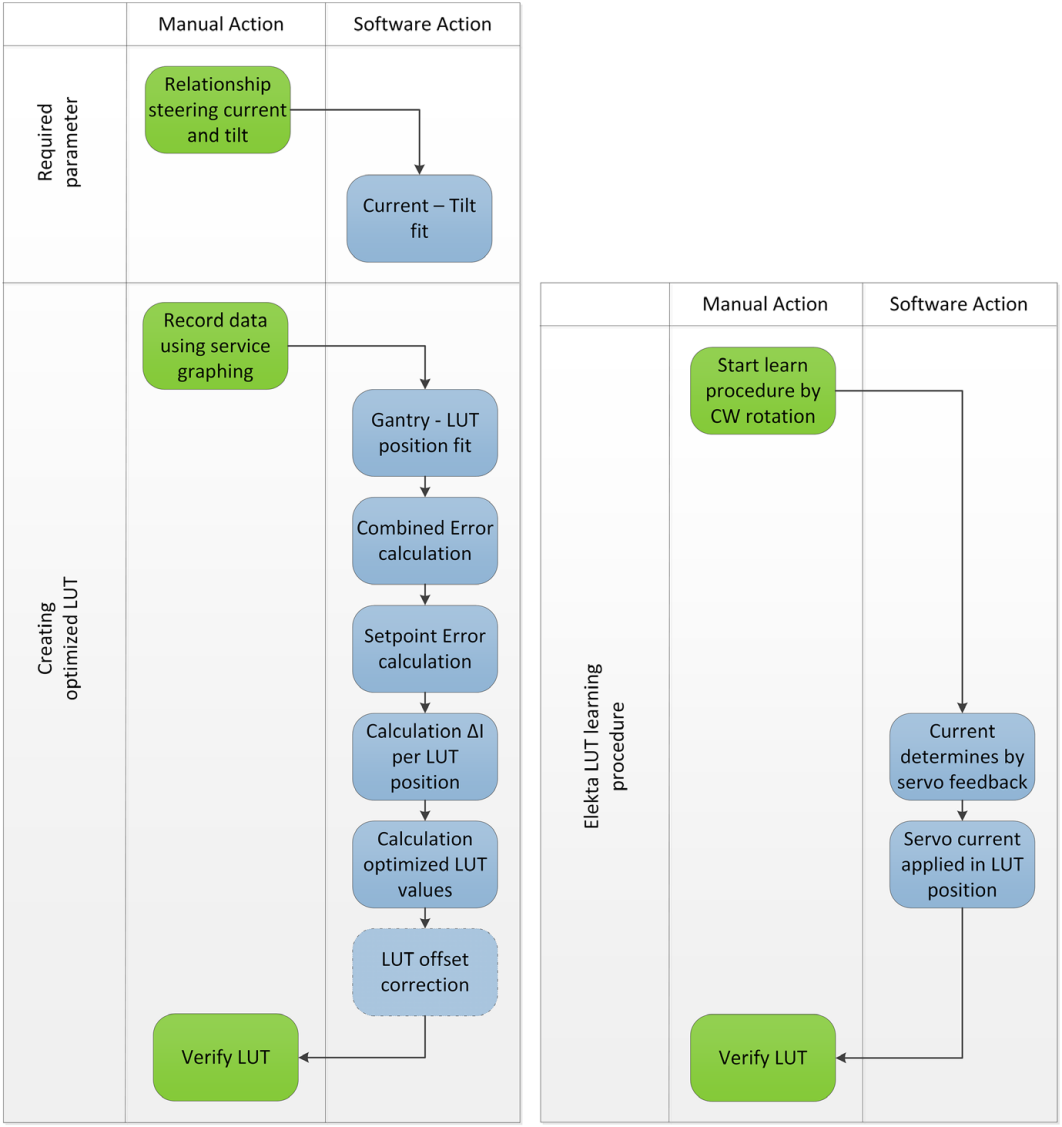
The left chart shows the order of this method to improve the Lookup Table (LUT). The steps are divided between manual actions which have to be done by an engineer on the linac and software processing actions. The right chart shows the basic learn procedure as per the Elekta manuals,^5^, ^9^ which has less steps and leads to a suboptimal LUT for counter clock‐wise rotation and without noise reduction.

The improved method to generate the beam steering LUT, requires more steps than the basic learn procedure as described in the Elekta manuals.^5^, ^9^ Figure 3 shows these steps for both procedures. The developed method, shown in the left chart, requires an additional input parameter which is the relation between steering current and beam tilt. The steps are further explained in the next paragraphs.

### 2.A Determination of the relationship between steering current and tilt

**Fig. 4:**
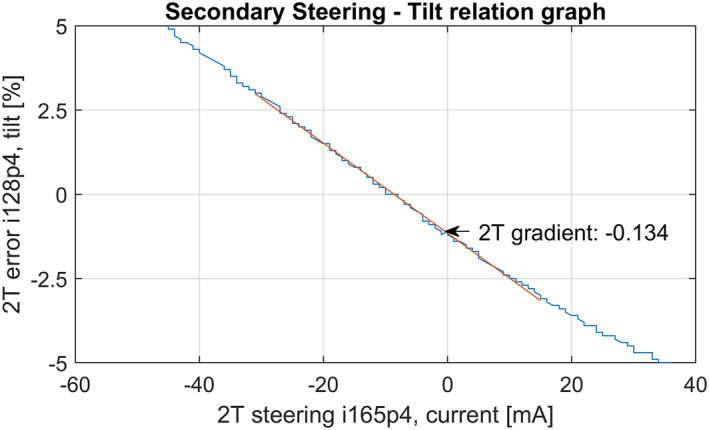
Example of the relation of the steering current and the cross‐plane (2T) error using the service graphing function of Integrity®.

The designed method is based on the relationship between the linac steering current and linac beam tilt. Figure 4 shows an example of the calculated relationship between the error and the current, using a linear fit. The calculated gradient in this example is −0.134 percent [%] per current unit [mA]. These gradient values of 2R and 2T are used to perform a forward estimation of the steering current to adjust the LUT for new optimized values. An incorrect relation will directly result in a proportional under‐ or overcorrection of the LUT. To minimize this under‐ and overcorrection effect, the total LUT correction is minimized as further explained in paragraph 2.3.3.

### 2.B Creating the main plot for LUT calculation

The input data for LUT optimization are the measurements of the actual tilt against the gantry angle. Other parameters included are the actual LUT value, actual LUT position and actual steering current value.

The recorded parameters for the Elekta SL25 accelerators are shown in Table 2. This dataset is recorded for both CW and CC gantry rotation data. Multiple measurement samples (between 4 and 7 samples) for each LUT position are recorded to prevent under sampling errors. A low gantry rotation speed is used to ensure high‐resolution data (this can done by a temporarily reduced Automatic System Unit (ASU) speed) and to reduce readout errors from the system delay in reporting the filtered tilt percentage. The 2R and 2T servo mechanism should be temporarily switched off to measure the beam tilt based on the Set value and LUT values.

**Table 2:** Items and corresponding part numbers which are required to make a gantry LUT plot.

	Item	Part	Description
X‐axis:	148	4	Gantry angle confirmed value
Y‐axis:	164	4	2RI control confirmed value
		136	2RI control LUT value on specific gantry angle, Integrity® 4‐
		211	2RI control LUT value on specific gantry angle, Integrity® 4+
		153	2RI control LUT position on specific gantry angle
	127	4	2R error confirmed value
	165	4	2TI control confirmed value
		136	2TI control LUT value on specific gantry angle, Integrity® 4‐
		211	2TI control LUT value on specific gantry angle, Integrity® 4+
		153	2TI control LUT position on specific gantry angle
	128	4	2T error confirmed value

Note

beam steering servos are switched off during data collection.

Measuring the data should start with the gantry outside the clinical range of gantry movement, for example, −181°, to ensure that the proper data is used within the limits of the clinical gantry angle. After beam on, it is recommended to verify visually on the Integrity window that the beam tilt (item 127 and 128) and uniformity (item 160) show stable values. This is to prevent transient beam effects from being incorporated in the collected data.

Before starting data collection, a waiting period of approximately 20 s before recording data is needed to have a stable beam without significant transient beam effects. The gantry can be rotate slowly back and forth (clockwise direction and counter clockwise) within 5 min.

The data were recorded using the service graphing function of Integrity®, with the maximum sampling frequency of 4 Hz. After collection, the data were exported and processed using a developed MATLAB® (The MathWorks, Inc., Natick, USA) program.

### 2.C Processing the data

#### 2.C.1 Determining relationship of LUT position and gantry angle

**Fig. 5:**
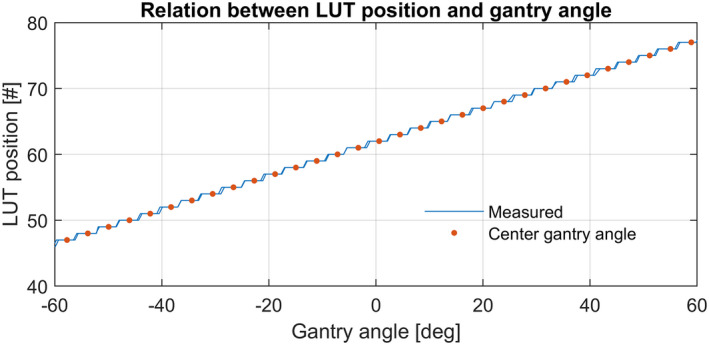
Plot of the relation between the gantry angle and the actual LUT position. Note that this varies slightly for each linac. For visibility a selective gantry angle range is displayed.

From the collected data, a linear fit between the LUT position and the gantry angle is derived. With this fit the mean gantry angle of each LUT position is calculated to overcome the discretization, as shown in Fig. 5. These gantry angles are linac specific as they are related to the raw potentiometer readout of the gantry angle measurement system.

Having the corresponding gantry angle per LUT position, the tilt per gantry angle to optimize the LUT can be determined. This tilt is derived from the recorded tilt percentage of the clockwise and counterclockwise data.

#### 2.C.2 Calculation the combined error for all gantry angles

**Fig. 6:**
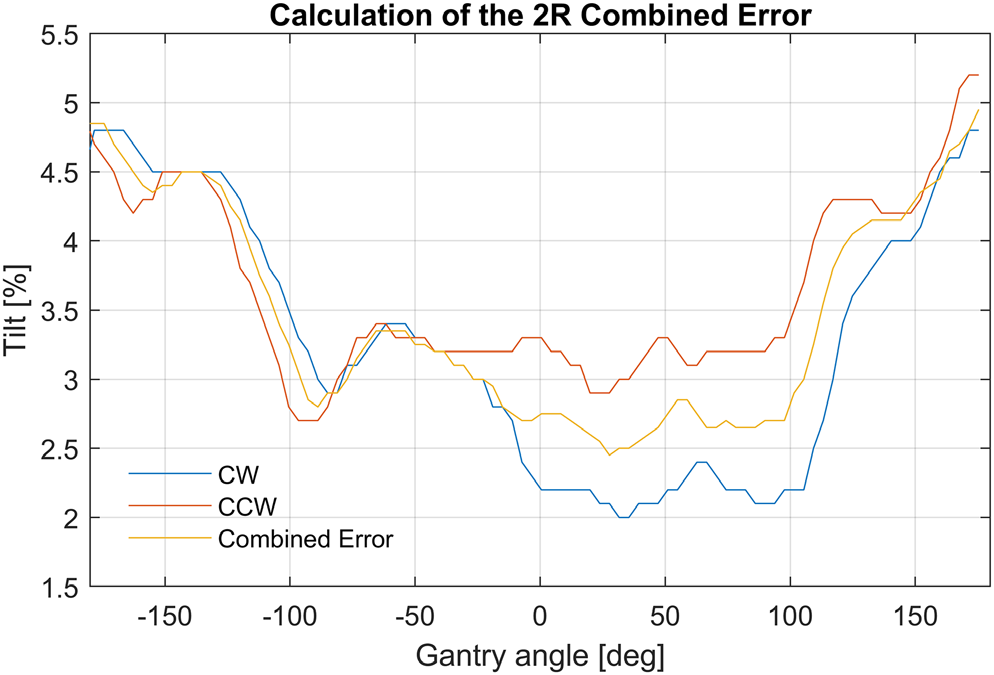
Example of the 2R error recorded using the Integrity service graphing function. This graph consists of the clockwise (CW) and counterclockwise (CCW) data, from where the Combined Error is derived.

As shown in Fig. 6, the recorded CW and CC tilt are not the same. This is due to the system delay and hysteresis. To obtain a single LUT for both rotation directions, a combined (average) error metric is derived based on the measured data of both rotation directions. The combined error is an intermediate step in the new LUT calculation and is determined in two parts. First, a phase shift is performed to compensate for a delay caused by readout latency and filtering. This phase shift is in practice determined as a delay of two LUT positions, which is roughly 8° of the gantry angle. Using a gantry rotation speed of 3° per second (approximately half of maximum) the delay is between two and three seconds. In Fig. 6 this phase shift is recognizable at −110 and + 110 degree. However, in the example shown in Fig. 2 where the CC and CW error curves do not have a clear determined correlation, an automatic shift based on the data itself is sensitive to inaccuracies. Generally, a delay of two LUT positions (almost 8 degree) appeared to be sufficient. Secondly, the combined error is an averaging of both lines (CW and CC) after the phase shift. The data of this combined error will be the input for optimization of the improved Lookup Table.

#### 2.C.3 Determination of the error setpoint for LUT correction

**Fig. 7:**
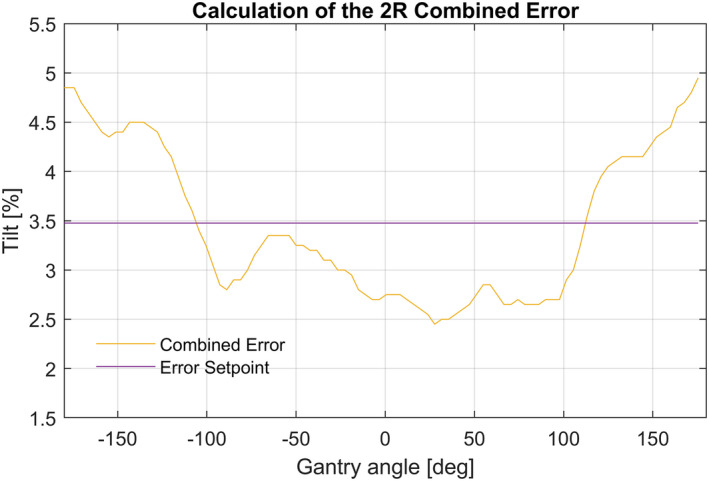
Shows the Combined Error which should be corrected to a value for all gantry angles. An offset is applied as well as a correction for the shape of the curve, in order to minimize negative side effects of under and overcompensation of the curve shape. This offset, the Error Setpoint, is calculated as the mean error over all the gantry angles.

After calculation of the combined error per LUT position, the error will be corrected by changing the current per LUT position. The combined error should be corrected to zero in the ideal case. To prevent overcorrection due to the multiplication of small errors, or a systematic error such as an incorrect Steering Current — Tilt equation (see Section 2.A), the LUT values are defined as deviations from a mean static error value. This static error, called Error Setpoint, is calculated as the mean value of the Combined Error. An example of the Error Setpoint as mean value of the Combined Error is shown in Fig. 7.

However, this correction of the LUT leads to an offset in the tilt, this offset in the tilt is corrected by the Set value.

The Error Setpoint is corrected to zero by changing the Set value according to Eq. (1) and Table 3.

**Table 3:** Overview of part numbers of 2T and 2R current control items.

Part	Description
1	Set value
4	Confirmed value/I
11	Calibration gain
136	LUT value, Integrity® 4‐
211	LUT value, Integrity® 4+

The Lookup Table can be used for other beam modalities as well (e.g., FFF or Electron energies).

The Set value is different for each energy configuration.

#### 2.C.4 Additional current calculated for each LUT position

**Fig. 8:**
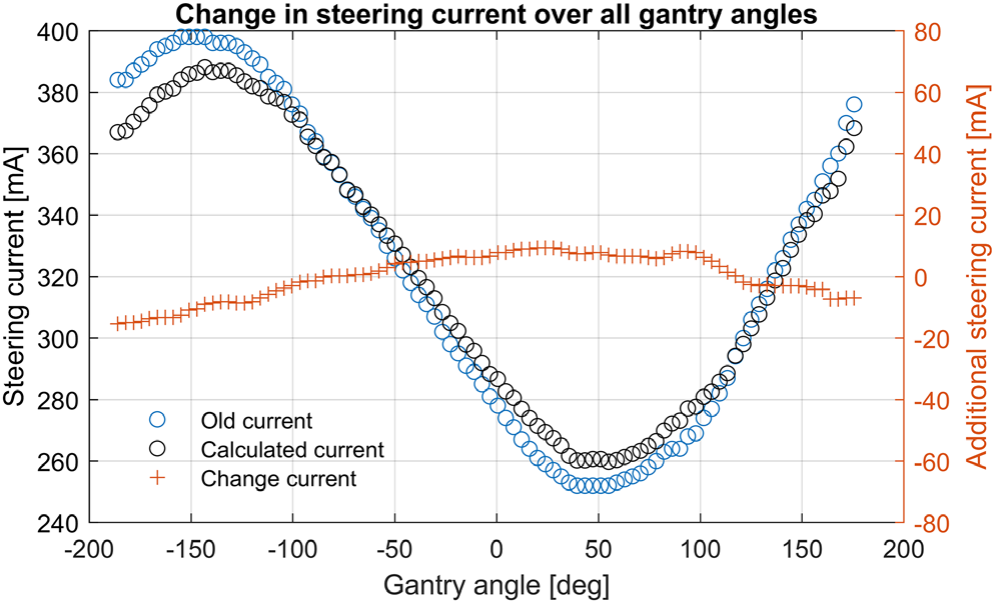
Example of the results of applying Eq. (3). The additional steering current (orange crosses) is added to the old current (blue circles) to give the newly optimized, calculated current (black circles).

Having derived the combined error per LUT position (B), the new optimized current (I) can be calculated with the actual current (A), the conversion between tilt and steering current (g), and the weighting coefficient (p), as shown in Eq. (3). Figure 8 shows an example of the old gantry angle dependent current, the additive current and the new calculated current.

(3) $$I_{\mathrm{steering}}=A+B*g*\frac{p}{100}.$$

The weighting coefficient, can be used for combining new and existing data. Anecdotally, this has led to more stable beam steering on a long‐term basis. A weighting coefficient of 100% should be used for a new installation or after a systematic change in the LUT is expected.

#### 2.C.5 Calculation new LUT values and smoothing the raw data

After calculating the coil current for each LUT position all new LUT values are calculated using the inverse of Eq. (1).

(4) $$LUT\;value=\left(I_{\mathrm{steering}}‐Set\;value\right)*\frac{2048}{calibration\;gain}.$$

Without any filtering, noise spikes in the recorded tilt data from the linac ion chamber, will be propagated to the calculated raw LUT. To smooth the LUT, a sinusoidal fit function is used consistent with the theoretically expected behavior due to the gantry rotation. Due to inhomogeneity in the magnetic field, the data is fitted with a function model consisting a sum of five sinusoids.

This fit smoothes the existing optimized LUT table which can then be resampled at the LUT positions. Furthermore, using a fit, the data in the LUT contains proper values for gantry angles outside the ±180 degree.

**Fig. 9:**
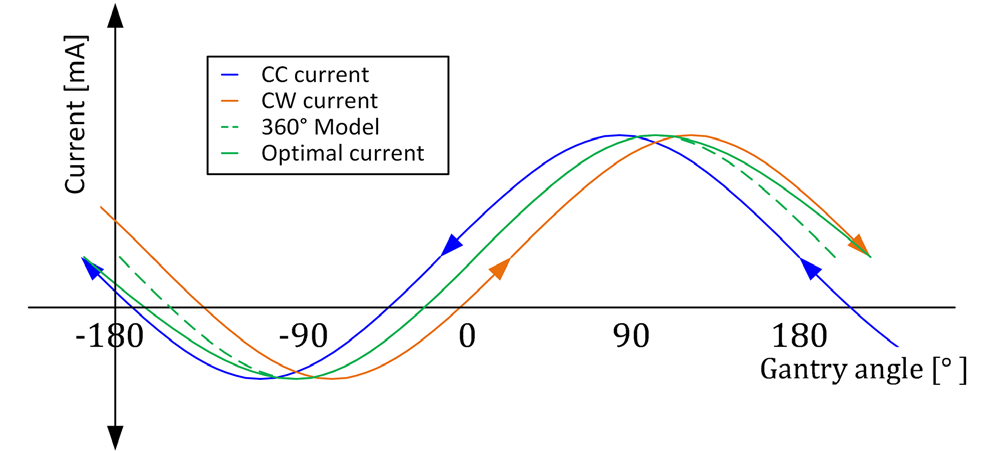
Shows the principle of required current to have zero tilt with gantry rotation CW and CC. In blue the required current in CC direction to have zero tilt. In orange the CW rotation. In dashed green the expected ideal current based on a 360 degree fit. In green there is visible that the ideal current of the LUT at the outer angles is closer to the rotation toward the outer gantry angles than opposite rotation.

In clinical use, the gantry can never rotate from outside the gantry −180 to +180 degree range. Instead of a theoretically expected sinusoidal model of 1 arc of 360 degrees, the combined model must match the appropriate single‐direction curve at each end, which is visually shown in Fig. 9. Therefore the optimal fit begins at gantry −180 degrees with the CC curve, makes a smooth transition to an averaged fit, and then ends at gantry +180 degrees matching the CW curve.

#### 2.C.6 Optional offset correction of the LUT

There are multiple valid solutions for Eq. (1) to reach the obtained current for each LUT position. Solving this equation does not influence the output of the equation, which means that these optional offset does not influence the steering current in clinical practice. Due to the effect that the steering current [I_steering_ of Eq. (1)] is the same. The reason to have multiple options is for the practical perspectives to minimize the required changes or to use a guideline to divide the current between Set Value and LUT from an logical or physics points of view with a preferred level of Set Value, LUT or combination of both. When all values of the LUT table are increased it will be compensated with the Set Value to obtain the same current per gantry angle.

Three different modes of LUT offset correction can be used. These modes are called “Normal,” “Min Max” and “Gantry zero.” Depending on the maintenance or QA purpose, which is described below, the appropriate mode is chosen.

Normal mode; The bare correction as described in paragraph (2.3.3). No additional offset correction on the LUT values is applied. LUT values and other energy configuration with the same LUT need to be adjusted as a minimum.

Min max mode; The LUT will be corrected such that the maximum value of the LUT becomes the same as the mathematical absolute minimum value:

(5) $$LUT\left(\max\right)==\left|LUT\left(\min\right)\right|.$$

Alternatively, one could calculate the LUT such that the integral over 360 degree is zero.

Note that in this case the Set Value gives some more information about the steering correction due to the fact that the gantry angle dependent correction is neglected in the Set Value.

Knowing the 2T current of opposite gantry angles should have in principle the opposite magnetic field correction, the Set Value does not include the magnetic field correction and should in ideal case, when perfect mechanical aligned, be close to zero. Due to an angle of 22 degree of the gantry arm, this method is not valid for the 2R direction, but only for the 2T direction.

Gantry zero mode; The LUT position which represents “gantry angle zero” is set to zero. In this case, the Setpoint value is the same as the initial current with LUT at gantry angle zero. Note that most QA and beam tuning is done in gantry 0 position. In that situation, it is desired from a practical perspective that the set value is equal to the running value when the servo mechanism is off. When the servo is on, it directly shows what the contribution of the servo mechanism is in that case.

**Fig. 10:**
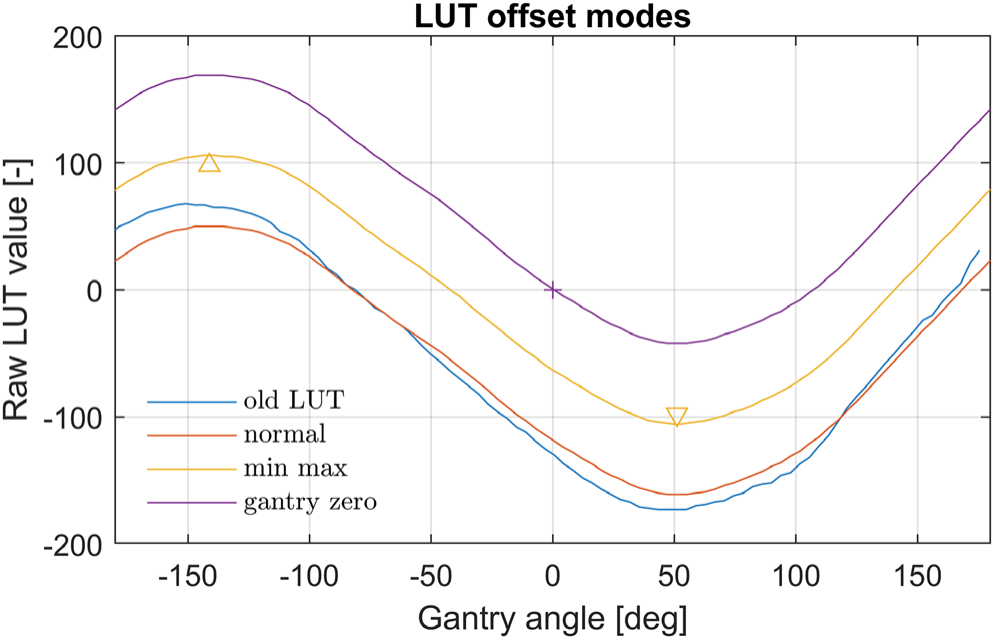
Shows the LUT offset correction modes. Red is the “normal” mode and is most close to the old LUT. Yellow is the “min max” mode where the mathematical absolute minimum value is equal to the maximum value. Purple is the “gantry zero” mode where the LUT contribution at gantry angle zero is zero.

These three modes are shown in the graph of Fig. 10. It is noted that a vertical offset in the LUT has no influence on the tilt when it is compensated with the set value. In this study the first mode is the default setting, which has minimal correction of the LUT.

## 3 RESULTS

There are many parameters to optimize beam steering of the Elekta traveling wave accelerator. The proposed method gives an automated forward procedure to reduce beam variation over all gantry angles.

**Fig. 11:**
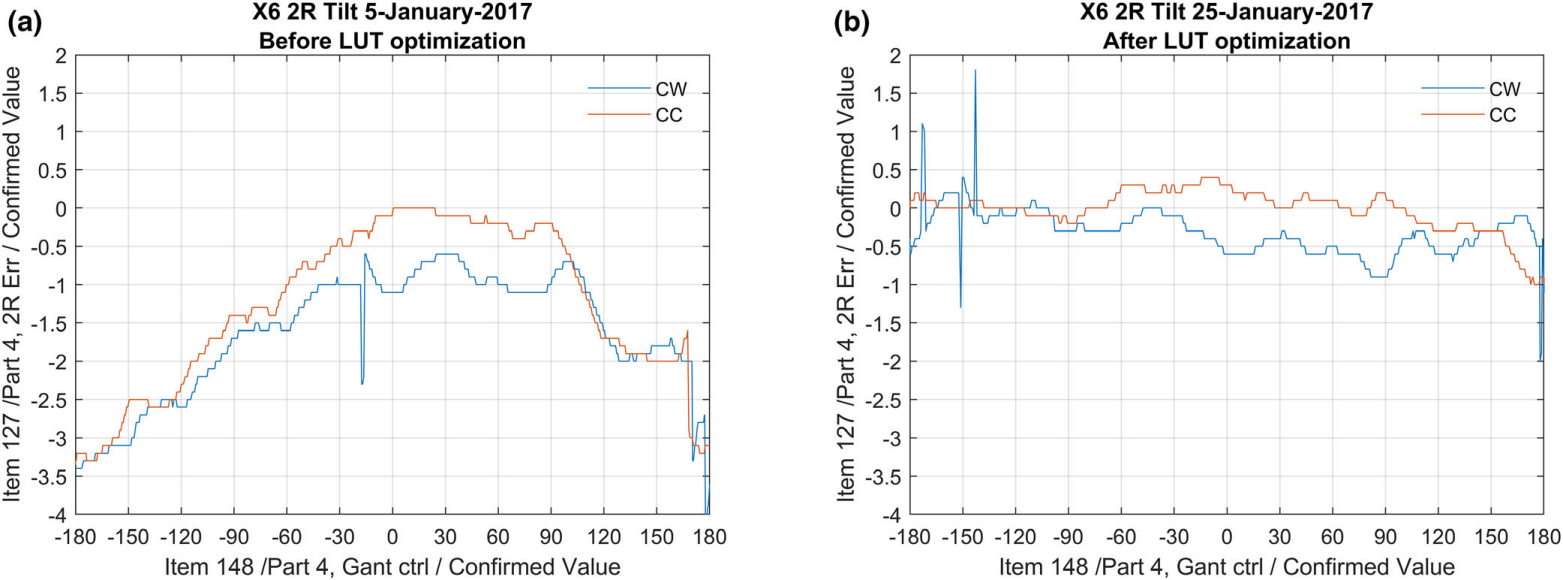
(a) A plot of the 2R error of a clinical machine with temporarily disabled servo mechanism. (b) An example of the tilt CW and CC after optimization as described in this article. The average error is smaller and mirrored around zero.

**Fig. 12:**
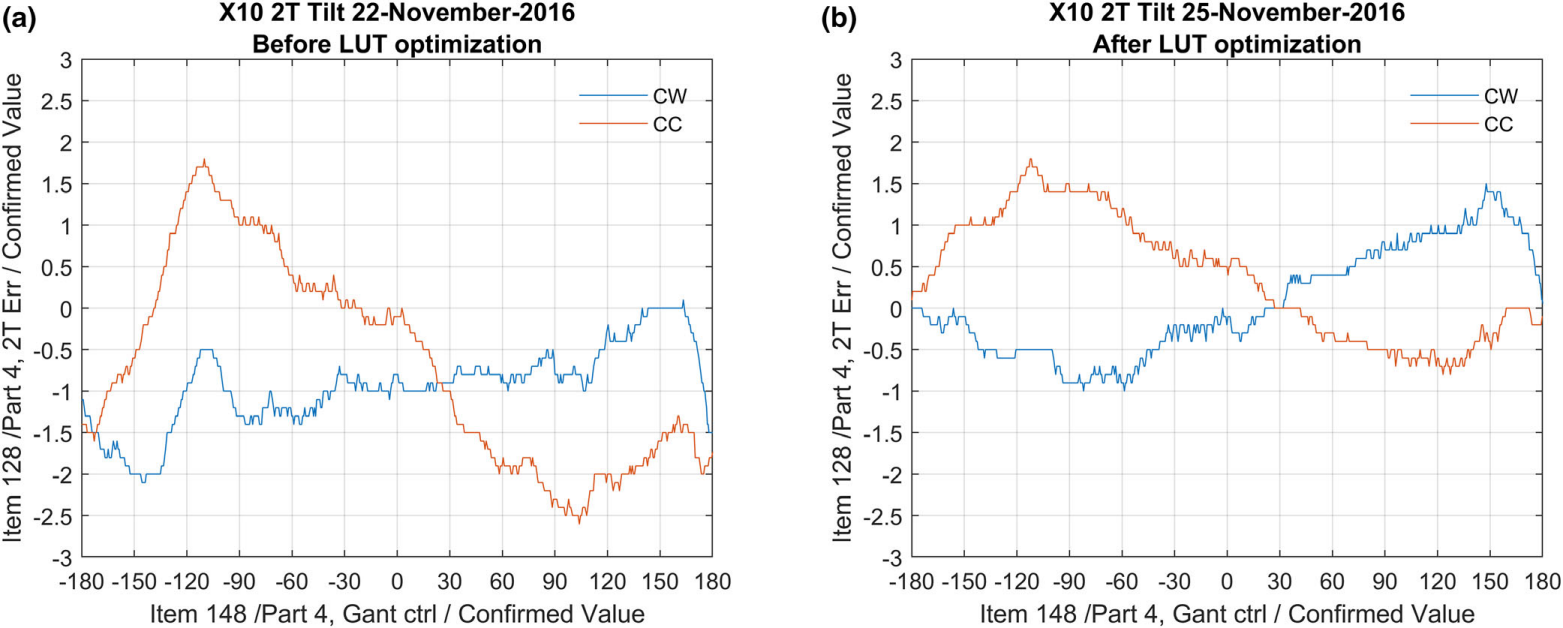
(a) A plot of the 2T error after making the LUT with the Elekta procedure. (b) An example of the method as described in this article, where the 2T error is mirrored around 0. The benefit of this method is that the range of tilt percentages is smaller which leads to a more stable beam with lower sensitivity for other influences (e.g., sub‐optimal Set Value, treatment modulations, etc.), and therefore less beam interruptions.

The result in terms of beam tilt are a more equal tilt for all gantry angles. Figure 11 shows an example of a 2R beam tilt optimization of a 6 MV energy and Fig. 12 an example of an 2T beam tilt optimization.

**Fig. 13:**
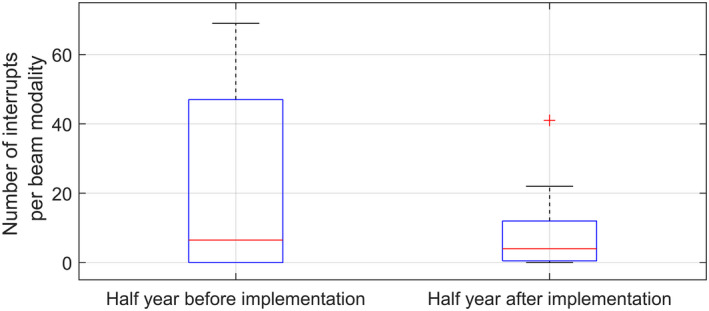
Comparison of the number of interruptions due to 2T, 2R, or Uniformity errors of the linac. This data comes from 10 linear accelerators with two energies (6 and 10 MV). Total number of interruptions is significantly reduced, from 393 to 165 in the 6‐month period, due to improvement of the Beam Steering Lookup Tables.

To quantify the performance of the linear accelerators a comparison of interruptions is done of the period before and after the LUT optimization using data from Elekta IntelliMax®. This comparison shows the amount of beam steering suspends over these two periods. The data shows that the new method reduces the beam steering related interruptions significantly on our linear accelerators. A reduction of 58% is achieved using this method, see Fig. 13. For comparison a period with a maximum of half year is chosen, except for the beam configurations where the 2T servo mechanism is switched on. This period is excluded in the period and shortened in the period other half year to have equal time periods. Total number of fractions with 10 clinical linacs and 2 flattened energies (6 and 10 MV) is 23 731 in the period before and 25 127 in the period after LUT optimization. Total number of interruptions is significantly reduced, from 393 to 165 in the 6 month period, due to improvement of the Beam Steering Lookup Tables.

## 4 DISCUSSION

The changes in power settings of the beam energy or changes in the environmental magnetic field of the linac cause changes in the optimal required 2R and 2T currents to get a symmetrical beam. It is important to verify the optimal beam energy settings before optimizing the beam steering LUT.

When the servo mechanism is on, the LUT is mainly functional for the initial current. This initial current without servo correction at beam start, is the time when most interruptions occur. These initial settings determine also the limits of the minimal and maximal control current. During treatment, the servo mechanism will lead to a reduction of interruptions of beam steering current. The reduction of interruptions due to the enabled servo mechanism was not included in this study.

Effects on the plan QA passing rates are not considered in this paper. In principle, a more symmetric beam corresponds better to the beam model of the TPS (Treatment Planning System) obtaining better plan delivery (higher gamma pass rates). However, regarding most of the delivered doses is after the transient effect of the beam, when the servo mechanism for 2T and 2R is active, a visible result according the gamma passing rates is expected to be negligible.

For a Flattening Filter Free (FFF) beam, where the 2T servo is disabled by the vendor, the initial values (LUT with Set Value) leads to direct influence of the beam alignment, which is outside the scope of this study. The real benefits can be found in the reductions of interruptions with their implications to the clinic.

After applying the new LUT, verification should be performed. Because the steering current is normalized to the current at gantry zero, the beam current value on gantry angle zero is not changed. QA must be performed at clinical representative gantry angles and not only on gantry angle zero. The gantry angle dependent steering should be verified when gantry dependent beam steering interruptions occur or, with every change of the linac power settings of the beam energy or, by changes in the environmental magnetic field (e.g., ramp of a nearby MRI magnet). To prevent beam steering interruptions it is recommended to verify the linac 2T, 2R and uniformity error on a six‐monthly basis. The internal ion chamber can be used if this chamber is appropriately calibrated. In this study the same parameters as described in Table 2 are monitored to verify that the error distribution is reduced.

If multiple modalities are using the same LUT, these energy configurations have to be verified as well.

The data collection are done with one specific dose rate. The Set Value should be verified with consideration for all dose rates, if the system is used for dynamical treatments with variable dose rates, such as VMAT.

## 5 CONCLUSION

This study shows an improved linac stability, with reduced beam interruptions, due to improved lookup tables. This reduces down‐time and reduces the chance of longer treatment times caused by beam interruptions. Shorter treatment times will reduce the chance and magnitude of intrafraction motion.

## AUTHOR CONTRIBUTION STATEMENT

A.A. van Appeldoorn conceived of the presented idea, developed the theory, and performed the measurements and analysis. J.G.M. Kok and J.W.H. Wolthaus verified the analytical methods, contributed to the design, and implementation of the research. J.W.H. Wolthaus supervised the findings of this work. All authors discussed the results and contributed to the final manuscript.

## Note

^1^ This servo part is always enabled for the 2R steering coils but disabled for 2T steering for the following energies 4MV X‐ray, all Flattening Filter Free (FFF) energies and all electron energies. Previously the other conventional X‐ray energies also had the 2T servo disabled, but this has been recently revised.^3^, ^4^
